# Comparing Health Workforce Policy during a Major Global Health Crisis: A Critical Conceptual Debate and International Empirical Investigation

**DOI:** 10.3390/ijerph20065035

**Published:** 2023-03-13

**Authors:** Ellen Kuhlmann, Jean-Louis Denis, Nancy Côté, Gabriela Lotta, Stefano Neri

**Affiliations:** 1 Clinic for Rheumatology and Immunology, Hannover Medical School, Carl-Neuberg-Strasse 1, 30265 Hannover, Germany; 2 Sociology of Health and Health Systems, Faculty I, University of Siegen, Adolf-Reichwein-Strasse 1, 57068 Siegen, Germany; 3 Département de Gestion, D’évaluation et de Politique de Santé École de Santé Publique, Université de Montréal, C.P. 6128 Succursale A, Montréal, QC H3C 3J7, Canada; 4 Département de Sociologie, Université Laval, Pavillon Charles-De Koninck, 1030, Avenue des Sciences-Humaines, Bureau 3469, Québec, QC G1V 0A6, Canada; 5 Department of Public Administration, Getulio Vargas Foundation, Av Nove de Julho 2029, São Paulo 01313-902, Brazil; 6 Center of Metropolitan Studies, Cidade Universitária, 109, São Paulo 05508-060, Brazil; 7 Department of Social and Political Sciences, University of Milan, Via Conservatorio 7, 20122 Milan, Italy

**Keywords:** comparative health policy, health workforce policy, health systems, social inequalities, COVID-19 pandemic, international comparison

## Abstract

Background: The health workforce is central to healthcare systems and population health, but marginal in comparative health policy. This study aims to highlight the crucial relevance of the health workforce and contribute comparative evidence to help improve the protection of healthcare workers and prevention of inequalities during a major public health crisis. Methods: Our integrated governance framework considers system, sector, organizational and socio-cultural dimensions of health workforce policy. The COVID-19 pandemic serves as the policy field and Brazil, Canada, Italy, and Germany as illustrative cases. We draw on secondary sources (literature, document analysis, public statistics, reports) and country expert information with a focus on the first COVID-19 waves until the summer of 2021. Results: Our comparative investigation illustrates the benefits of a multi-level governance approach beyond health system typologies. In the selected countries, we found similar problems and governance gaps concerning increased workplace stress, lack of mental health support, and gender and racial inequalities. Health policy across countries failed to adequately respond to the needs of HCWs, thus exacerbating inequalities during a major global health crisis. Conclusions: Comparative health workforce policy research may contribute new knowledge to improve health system resilience and population health during a crisis.

## 1. Introduction

The health workforce is the “heart and soul” [1] of every healthcare system and is central to population health. The COVID-19 pandemic put a spotlight on this connectedness, which threatened both the health of individual people and the functioning of healthcare systems; this was observed even in countries with comparatively high resources and public health expertise [2,3,4]. The crisis reminded us that ‘care of the patient requires care for the provider’ [5]. This suggests, that “unpacking health policy and population health” must consider the outcome of pandemic policy in relation to the healthcare workforce (HCWF) and how it affects the individual healthcare workers (HCWs).

We argue that failure to adequately prioritize the HCWF’s needs during a major global health crisis puts the health and well-being of individual HCWs and the provision of healthcare at risk. It weakens the implementation of universal healthcare coverage (UHC), a priority policy goal of the United Nations’ Sustainable Development Goal (SDG) 3 ‘Health’ [6]. The crucial relevance of HCWs calls for health systems to be held accountable for pandemic preparedness and protection of HCWs, including the prevention of inequalities and better support for women and migrant HCWs who might be more vulnerable to the impact of the crisis [7,8]. However, health policy tends to prioritize material resources over human resources for health. The lack of attention to the mental health of the HCWs even in the fourth year of the pandemic, an increase in burn-out among HCWs, and an overall exacerbation of inequalities are some of the examples that illustrate the threats of policy failure [9,10,11]. 

Research is mirroring the lack of attention to HCWs and thus reinforcing policy failure. Health workforce policy has largely been ignored or reduced to “faceless numbers” [1] of HCWs, serving the health labor market forecasting and planning exercises of researchers and policymakers. The person behind every HCW [12] and more complex issues of health workforce governance are rarely explored more systematically. Healthcare workers are a highly diverse group of professionals. In most countries, doctors and nurses are the two largest groups of HCWs and a very heterogeneous group of carers increasingly also plays an important role. These are professions, occupations, and individuals with very different needs; they also strongly vary in relation to their gender and race/ethnic composition. The health workforce is shaped by strong unequal distribution of power and resources. These inequalities intersect in different ways and have been exacerbated during the COVID-19 pandemic [10,13,14]. For instance, a WHO report based on data from Germany, Italy, and Spain reveals that confirmed COVID-19 cases among female health workers are two to three times higher than those observed among their male counterparts [15].

Growing gender inequality is a global problem that is affecting all women and girls [8], but most seriously affects migrant women [14,16]. HCWs also strongly vary in relation to their stakeholder roles. Some health professional groups are powerful corporate players integrated into the policy processes (e.g., physicians), or acting as external stakeholders furnished with the power of policy expertise and leadership [17]. Others lack political and scientific power, such as less well-educated and resourced groups, typically comprising informal carers, and in many systems also parts of the nursing profession.

No health system typology exists that would help to thoroughly consider the health workforce as a system indicator [18,19,20,21,22]. The pandemic has added further problems. Different health systems often responded similarly, thus challenging comparative research far beyond health workforce policy. As Greer and colleagues revealed in their comprehensive collection of international case studies, “coronavirus politics” do not sit easily with the conceptual frameworks of comparative policy [23], and this was also confirmed by other comparative research [2,7,24]. 

This study puts HCWs center stage. We seek to explore the substance of health workforce policy across different levels of governance and illustrate why it is an important dimension of comparative health policy. The situation of HCWs during the COVID-19 pandemic serves as a policy arena for our analysis. As Liu et al. state, the pandemic was a kind of natural experiment that brought existing policies to the test [24]. Four countries have been selected as illustrative cases, comprising Brazil, Canada, Italy, and Germany. This selection represents different healthcare systems in high-income to upper-middle income countries; it should be noted that we refer to Brazil under the President Bolsonaro government (until December 2022). Most importantly, we move beyond typologies and consider variations in the composition of HCWs, the geo-political contexts, and the COVID-19 policies; a similar approach was applied to previous research and was proven to be useful [2,7,8]. We expect that these differences also affect to some extent health workforce policy and governance. 

The aim of this study is two-fold: to critically discuss comparative health policy and system research and introduce an integrated multi-level governance approach that sets the focus on the human resources for health, and to contribute empirical evidence to HCWs’ protection and the prevention of inequalities during major public health crises.

## 2. Methods

### 2.1. Theoretical Background: Aligning Health Workforce Research and Comparative Health Policy

Health workforce research is an emergent field, involving various disciplinary approaches and stakeholders. There is no uniform and coherent definition; the substance and priorities may vary between disciplines and stakeholder groups. However, a common denominator is an attempt to strengthen health workforce issues in health policies and systems and to improve empirical data and monitoring systems. This includes classic research topics of health labor markets, planning, education, and regulation, as well as raising awareness of the needs of HCWs (micro-level) and improving knowledge of organizations, stakeholders, and health policy and systems. Characteristically, health workforce research aims to move beyond academic debate and make an impact on policy and practice [25].

For many years, health workforce policy and research were primarily driven by the WHO and World Bank and other international organizations, focusing on low-income and middle-income countries (LMICs) [1,26,27]. The WHO has greatly helped to move HCWs and health workforce policy issues higher up on the global health agenda [3,28,29]. 

Many high-income OECD countries, including Germany and Italy, have long ignored health workforce policy and limited their efforts to basic issues of regulation, education, and planning. In contrast, HCW research and policy are more advanced in Canada [30] and Brazil [31]. Dussault highlights that no action was taken at the European level until 2008. Notably, health workforce policy is under the authority of the nation-states [27]. In this situation, the European Union (EU) policy mainly acts via education and health labor-market policies [32]. The health workforce is poorly represented in the EU research agendas; existing projects and funding programs tend to focus on HCW planning and networking issues, while policy and governance are generally weak. 

Across countries, health workforce policy and research are poorly prepared to respond to the growing shortage of HCWs and the new challenges of the COVID-19 pandemic. There is also a serious need for improving HCW monitoring and data sources, in particular in relation to the standardization and comparison of data [33]. In this situation, health workforce research benefits from its multi-disciplinary nature and the connection of different concepts. Three approaches are especially useful: sociology of professions, organization studies, and comparative health policy.

- Sociology of professions can help us better understand the complexity of health policy. It can provide in-depth information on the stakeholder groups [34] and analyze actor-centered developments at the micro-level [35];
- Organizational studies highlight the operational dimensions of governance and leadership issues, including the role of professional actors [17]. The concept of “street level bureaucrats” is another useful framework for comparison, which highlights the connectedness of professions and organizations [36];
- Comparative health policy may explore institutional conditions and macro-level policy developments. There is no common approach to cross-country comparison and existing typologies are highly diverse, yet most authors refer to three basic categories, including governance (or regulation, in previous classification schemes), finance, and the provision of healthcare (for an overview, see [18] (pp. 6–14)).

Governance theories are interlinked with each of these approaches and are most helpful for comparative exercises. Following Greer and colleagues: “Governance is how societies make and implement decisions… everything from the ability of policy-makers to take evidence-based and relevant decisions to their ability to implement policies and create alignment between different actors” (p. 6) [37]. Governance approaches may connect processes and actors systematically across the different levels of governance. Next to the regulatory dimensions at the macro level, governance includes operational dimensions at the organizational level and the stakeholders involved [34,38].

COVID-19 has added urgency to the HCW policy debate. It has revealed that we must look at different levels of governance, that may impact differently on the health workforce and therefore create a range of specific policy needs. For instance, a global lens is particularly important to respond to HCW migration and mobility flows, which reinforce existing inequalities between health systems and may increase the “care drain” in LMIC countries and within the EU [39,40]. At the micro level, the pandemic has reminded us that “care of the patient requires care for the provider” [5]. There is growing research evidence that the COVID-19 pandemic has reinforced stress and burn-out symptoms in HCWs [41].

Health systems across the globe are in need of strengthening resilience [42]. Building back better after the pandemic must include efforts to improve the resilience of the health workforces [2,4,7]. This calls for better data and research, and for more effective policies, all of which would greatly benefit from comparative approaches. Researching health workforce policy comparatively may improve knowledge exchange and support countries investing in their HCWs and strengthen resilience.

### 2.2. Introducing a Conceptual Approach for Comparative Health Workforce Research

Healthcare has always been a highly controversial policy field [18,43] and health workforce policy mirrors the challenges [44]. The vast majority of theoretical and empirical comparative health policy investigations do not (or only marginally) consider the health workforce. Wendt introduced a complex health system typology including HCWs as one indicator [22,45]. Böhm et al. revised this approach and identified hierarchical relationships between three different dimensions; more recently [19], Reibling et al. added further complexity to the model [21]. Characteristically, these approaches consider HCWs primarily as numbers and health labor-market items in relation to other health policy items, but do not grasp the complex role of the health workforce and the person behind every HCW [12]. Blank et al. [18], in their international comparison, applied an exploratory approach and added the health professions/HCWs as a policy field of its own. This approach provides in-depth information but needs further investigation to better understand the connections and system conditions. 

The pandemic has more generally questioned existing categories and further complicated the business of comparative health policy. This has been shown for different macro-level categories related to healthcare systems. For instance, in relation to the “regime types”, Greer et al. conclude from an international comparison of “coronavirus politics”: “Regime type was not a particularly conclusive variable in our findings… Broadly, we found similarities across regime type, and distinctive paths within regime type, more promising than regime types only” (p. 27) [23]. Typologies of healthcare systems embody similar problems, as health workforce research revealed. A comparative study of health system resilience and health workforce capacities in high-income countries during the pandemic (Canada, Austria, Denmark, Germany, and the Netherlands) confirms a lack of a coherent institutional pattern [2]. Similarly, an assessment of EU workforce policy found a convergence of health workforce policy priorities during the pandemic, as well as similar governance gaps across different types of health systems [7]. Comparisons of HCWs acting as “street-level bureaucrats” during the pandemic pointed out that HCWs had similar needs for support during the pandemic and that different countries responded in a similar way to the new situation [14,36].

Against this backdrop, we suggest a multi-level health workforce governance approach, which was first developed in the European context [38] and has also proven to be useful in Canada [30]. The conceptual framework comprises system, sector, organizational/occupational, and socio-cultural dimensions; the latter focuses on gender equality and migrant HCWs (in the Brazilian case, on racial inequalities). It considers the substance of governance across the hierarchical (transnational, macro, meso, micro) levels. The approach is sufficiently comprehensive to grasp the complexity of HCW governance and, at the same time, be flexible to respond to system-specific conditions. The framework informed the development of an EU health workforce research tool and was recently amended to include HCW policies during the COVID-19 pandemic [7]. 

Table 1 introduces a tool and selection of assessment categories that will facilitate comparison and guide the presentation of our four country cases. The framework was developed for health workforce policy and research and pays particular attention to multi-level governance and integration across sectors and levels.

### 2.3. Gathering and Analyzing the Material

We empirically investigated two comparative approaches: a descriptive comparison based on health system indicators and quantitative items, and a qualitative explorative case study design. We drew on secondary sources (literature, document analysis, public statistics, reports) and country expert information. The first approach aligns classic health system categories with health workforce and COVID-19 related items, as shown in Table 2 below. The second approach is guided by the multi-level governance tool (Table 1) that we introduced in the previous section. A step-by-step procedure was applied: the country case studies were prepared individually by the respective country expert(s), discussed in the team of authors, and subsequently revised and condensed and again discussed until sufficient coherence was achieved for the comparative analysis (based on qualitative methodology [43]). In addition to the written country cases, the major results are summarized in Table 3. 

## 3. Results

### 3.1. A Quantitative Descriptive Health System-Based Approach: Comparing Brazil, Canada, Germany, and Italy

We begin with briefly introducing our sample. Table 2 provides an example of a typical comparative descriptive approach informed by major health system categories and basic quantitative indicators related to the research topic, based on expert information, published reports, and public statistics (mainly OECD, national government for Brazil). We refer to the first two major waves of the COVID-19 pandemic, from the declaration of a pandemic in early 2020 until the summer of 2021.

### 3.2. An Explorative Qualitative Case Study Design: Comparing Health Workforce Governance and COVID-19 Policy in Context across Countries

#### 3.2.1. Brazil

Brazil has a Unified Health System (SUS), conceived during the 1980s based on the country’s democratization process. The SUS is guided by three principles: the idea of a universal and comprehensive right to healthcare services organized into different levels of care; decentralization, which makes all federative entities responsible for service provision; and social participation in health policies. The SUS works within a federative logic, in which the federal government, states, and municipalities are co-responsible for the finance, regulation, and implementation of services. 

Free and universal healthcare is organized into three sectors: primary, secondary, and tertiary care. Primary care is responsible for prevention and promotion and is offered in health clinics. Secondary care provides specialized services in clinics, while tertiary care is focused on hospitalization and more complex health services. The levels of care are integrated and offered cooperatively between states, municipalities, and the federal government. The federal government is responsible for the national coordination of the SUS, including policy development, planning, financing, and control. State governments have responsibility for regional governance, coordination of some strategic programs, and the provision of specialized and tertiary services. The 5570 municipalities are responsible for implementing primary care policies and part of secondary care policies. The system’s financing is also governed by the three federative entities, which are obliged to allocate a minimum amount of 15% to 22% of the budget to the health system [48]. 

When observing the size and vast experience of the SUS, including in previous public health emergencies, it was expected that Brazil would respond well to the COVID-19 pandemic. However, in the spring of 2021 the country was ranked as the worst case in the world with nearly 600,000 deaths [49]. This raises the question: what went wrong? The explanation is directly related to the position of past President Bolsonaro and the health ministry. The President was a denialist of the pandemic. From the beginning, he tried to minimize the risks, creating a supposed conflict between health measures and economic measures. He was against isolation policies and attacked governors and mayors who implemented policies of physical distancing, mandatory use of masks, and quarantine; he also opposed vaccination and postponed the purchase of vaccines for months [48,49]. Current investigation shows that vaccine purchases were made through a vast corruption scheme. 

The effects of the President’s speeches go far beyond rhetoric: they destabilized the SUS and hampered coordination. The health ministry did not exercise its coordination role in relation to the funding of pandemic policies and the regulation of the health workforce. This created a federal conflict, as mayors and governors were facing the pandemic without support and resources and had to take individual action. In addition, the President’s denial speeches had a practical effect on the health workforce. Examples are the pressure on HCWs to adopt ineffective medications and the strengthening of the anti-vaccine discourse [48].

Concerning sectors, the SUS is one of the largest public health systems globally and the health workforce comprises more than 6 million HCWs. However, there were still severe shortages due to a lack of specialized physicians and an unequal distribution of HCWs across the national territory. Although access to healthcare has grown considerably since its creation, the SUS still has problems with the quality of services, which are strongest in specialized and hospital care. Brazil is one of the most unequal countries in the world, and the SUS reflects these inequalities. Despite creating several policies that seek to address inequalities, there are still many inequalities in relation to both access to care and quality of services, comparing region, race, and gender [10,13,14,16].

In relation to the organizational and professional dimension, the health workforce had to work during the pandemic without resources within a context of conflicts and ambiguities [14], facing a stressful environment, and increasing demand from patients. Surveys carried out in April 2021 showed that more than 50% of the HCWs did not receive adequate PPE, 70% did not receive training, 65% did not feel supported by the government, and 80% had mental health issues [13,14]. Brazil has the largest number of nurses who died of COVID-19 in the world. In August 2020, almost half of the nurses who died from COVID-19 were Brazilian [50]. 

Concerning socio-cultural dimensions, these conditions further exacerbated inequalities, although they jeopardized the entire health workforce, Research evidence shows that women and black HCWs were most strongly affected by the pandemic [51]. Inequalities are also evident between professions. More powerful and better-paid professions, such as physicians, are in a better situation than less institutionalized professions, such as community HCWs [13]. The Brazilian case illustrates how political denialism and missing coordination threaten the HCWs, even in a well-established and experienced public health system [14]. 

#### 3.2.2. Canada

In Canada, healthcare services are mostly under the responsibility of provincial (sub-national) jurisdiction and financed through income tax payments (Beveridge model). Universal health insurance guarantees the coverage of medical and hospital care, other health and social care services are at the discretion of the Province. Notably, the system is labelled “health insurance” but is not financed by insurance contributions but mainly through taxes. The federal government plays a relevant role, particularly by using its spending power to uphold national standards for the coverage of medical and hospital services. Yet Provinces and Territories enjoy high autonomy; they are obliged to provide about 80% of total funds, plan health services, and decide on governance arrangements. Canada comprises ten Provincial healthcare systems and three Territorial healthcare systems with their organizational arrangements and priorities and should therefore not be considered as single healthcare system [52]. However, the health workforce shortage is considered a national crisis with more than 100,000 vacant jobs in healthcare [53]. 

From a system perspective, the two largest Canadian provinces, Ontario and Quebec, were severely impacted by the pandemic from the beginning, seeing dramatic death tolls in the most vulnerable segments of the population, namely frail elderly residents of long-term care (LTC) homes [54]. Alberta had considerable trouble confronting subsequent waves of the pandemic; the healthcare system was overwhelmed by the fourth wave of the pandemic. Like other jurisdictions, all Canadian Provinces struggled to ensure health system capacity to accommodate surges of COVID-19 cases and variants, which significantly reduced the ability to respond to non-COVID-19 care needs. British Columbia seemed to be better equipped to face the pandemic and to limit its detrimental effects on population health and the delivery of care [55]. 

The federal government played a key role in the procurement of vaccines, the supply of personal protective equipment (PPE), and ventilation equipment. During the first wave, the military forces were deployed at the request of two provinces, Quebec and Ontario, to help mitigate workforce shortages in the long-term care (LTC) system. Alberta contemplated this option during the fourth wave. Provincial governments and professional colleges also responded to the new health workforce needs and promoted several health system adaptions; for instance, the establishment of a remuneration scheme to encourage the development of tele-consultations in medicine and skill-mix changes/task-shifting efforts to increase the scope of practice of nurses, pharmacists, and other health professionals.

From a sectoral perspective, the system is hospital-centered with major quality of care and safety issues in the LTC sector, as underlined by a recent report commissioned by the Royal Society of Canada [56]. Access to PPE outside the hospital sector was severely limited with dramatic consequences; for example, LTC workers in Quebec faced an approximately 10 times higher risk of contracting COVID-19 compared to the general population [30]. 

Considering professions and organizations, the nursing profession is emblematic of the health workforce crisis. A report on the future of the nursing profession estimated that 27.1% of nurses plan to leave the profession [57]. Compulsory overtime work and stressful working conditions are considered as the main reasons for job resignations. Other professions or occupational groups, such as healthcare assistants, also face severe shortages. Provincial governments have taken several efforts to respond to health labor-market shortages and the growing demand for HCWs, including fast training and an increase in payment. However, no national database exists and effective health workforce policies that might respond to the health workforce crisis are lacking. There are also signs that middle-management positions in healthcare are difficult to fill, which might reveal more general problems of leadership in healthcare.

Health organizations and HCWs have done their best to compensate for health system problems. This might help in voicing for and achieving better recognition of all HCWs in future. Health organizations and HCWs also developed new approaches to the provision of care and improved collaboration across professions, which might be further developed for pursuit in the aftermath of the pandemic [53]. At the same time, the pandemic has revealed important health system problems [30], including important segments of the health workforce that appear to be undervalued, such as, for instance, nurses and middle managers. 

In relation to socio-cultural effects, the policy deficits affected women and migrant HCWs most; public discourse tends to reproduce prevailing gender inequality in relation to the valuation and recognition of work [30]. Gender often intersects with racial inequality. During the crisis, health workforce policy sought to quickly expand the mobilization of asylum seekers. There are high numbers of recent immigrants and members of racialized groups, especially in urban areas. These groups faced higher risks of contracting COVID-19 at work due to poor protection and work conditions, and at home due to poor housing conditions [30].

#### 3.2.3. Germany

Germany counts as a welfare state with a classic Bismarckian-type social health insurance (SHI) system. The system is decentralized and federalist and based on participatory governance. It is strongly shaped by corporatism with the medical profession as a powerful actor integrated into the policy process, creating complex negotiation processes between stakeholder groups and between national and regional levels of policymaking. The government has delegated power to a joint self-governing and self-administering body, the Federal Joint Committee, which is based on the SHI sickness funds and SHI Physicians Associations as the two main pillars and the Hospital Society. In healthcare policy, the states (*Länder*) have strong regulatory powers with some centralized regulation and coordination efforts, which were reinforced during the pandemic. The healthcare system is well-resourced and financed primarily via SHI contributions [58,59]. Health workforce staffing levels are among the highest in OECD countries, yet a shortage of all groups of HCWs is a serious problem and recruitment of migrant HCWs is on the increase [18]. Germany faced severe waves of COVID-19, but was less strongly hit than the neighboring countries and lockdown policies were more moderate [60]. 

The institutional conditions of federalism, decentralization, and corporatism shape the politics and the substance of HCW governance [7]. At the system level, we find well-resourced, but poorly integrated, health and education systems with overall weak public-health institutions. The Federal Health Ministry took action to increase the capacity of public health [61] and to introduce financial compensation and rewards. A compensation scheme for office physicians was established and a budget was provided for a financial bonus for nurses (single payment). Vaccination policy is another example of centralized efforts in an otherwise decentralized system. The vaccination prioritization was decided centrally, while the management was delegated to the states. Some efforts to improve HCW surveillance and create a national database and monitoring system were undertaken by the German Public Health Institute (Robert Koch Institut) [62]. However, data sources and information on the protection of HCWs are still poorly developed, although quality data are available.

At the sectoral level, Germany is known for its large and over-resourced hospital sector [58]. In contrast, the public health sector is small and poorly developed; during the first wave of the pandemic, a new program was introduced aiming at increasing staffing levels and strengthening public health competencies [61]. However, public health leadership is still lacking or poorly developed. Furthermore, HCW surveillance and protection strongly varied between sectors; personal protective equipment (PPE) and surveillance measures are strong in hospitals, but weaker in the LTC sector, affecting especially nurses and carers. Research evidence suggests that the COVID-19 infection risk of hospital workers might be largely in the range of the general population, as documented for a large academic hospital [63].

The meso-level, the organizational conditions, played a key role in the protection and preparedness of HCWs, which also explains the significant sectoral differences. Innovation and change were often stronger on the organizational level than on the system level, but the outcomes were more diverse and sustainability may be more hampered. The skill mix and professional and sectoral collaboration improved in some areas and new ad hoc solutions were established; some up-skilling was also observed, e.g., for healthcare assistants, and public health workers [2]. However, there are currently no signs that this may change the occupational field and/or the hegemony of the medical profession. HCW protection was overall strong but focused on PPE, surveillance, and vaccination, while little if any attention was paid to the mental health needs of HCWs, despite an increase in stress and uncertainties during the COVID-19 pandemic [7]. 

Concerning social inequalities, gender equality is defined by law and policies are in place in the public sector (similarly in many private organizations). However, little action was taken to mitigate the gender bias of the COVID-19 pandemic, affecting women and girls most [8,15,51]. There is an overall lack of recognition of female expertise at all levels of policymaking and also in the media; few women are in high-level decision-making bodies, especially during the first wave of the pandemic. In relation to migrant HCWs, specific policy agreements were in place to facilitate cross-border HCW mobility within the EU when borders were closed, especially bi-national agreements in border regions. There was a strong interest in maintaining migrant HCWs in the country, but health policy did not take care of the needs of foreign HCWs during the pandemic. There was some support at the organizational level to maintain or attract foreign HCWs, but this was highly diverse and did not achieve policy changes.

#### 3.2.4. Italy

The Italian healthcare system is a highly decentralized, Beveridge-type National Health Service (NHS) instituted in 1978, when it took the place of the previous Social Health Insurance system. The 20 Regions enjoy most of the powers and responsibilities in the organization and regulation of health services; the central government has control over most financial resources and has preserved major competences in the regulation of many kinds of healthcare resources, including staff [64]. During the pandemic, the national government and Regions had to manage the emergency together, alternating cooperation and conflict but trying to develop new forms of coordination [65]. Primary care is provided by GPs and pediatricians, who are independent workers contracted by the NHS. A total of 75–80% of hospital beds are in public hospitals, while the share of private providers (for-profit and non-profit) is higher, though still small, in specialist outpatient activities. Public health was at the core of the NHS 1978 regulation, but it has become a small and marginal sector over the years. This helps explain why public health services were able to provide a limited contribution to containing the COVID-19 contagion. 

Taken as a whole, NHS staff employed about 650,000 persons from 2018 to 2019. Between 2009 and 2018, its staff decreased by 6.5% [66,67]. The staffing levels are considered as low, however this is true for nurses but not for physicians. However, there is a maldistribution of physicians among specialties, as well as regional inequalities and shortages in many areas. Medical dominance has always been relevant, but physicians are external experts in the policy process with limited powers. 

Italy was one of the first countries in the world hit by COVID-19 and strongly suffered from the pandemic, especially in the first wave (mid-February until May 2020). A strict lockdown adopted in those months was motivated by the need to prevent the NHS and its hospitals from a collapse, because of the high number of patients [68]. Staff shortages were considered one of the main causes of the difficulties in tackling the COVID-19 emergency. Three kinds of measures were adopted at the national level [69]. Firstly, extraordinary recruitment plans were approved. Secondly, a national professional qualification exam for physicians was abolished, which previously was compulsory to enter the medical profession. Thirdly, Regions and NHS providers were allowed to assume extraordinary decisions aiming to extend working time and flexibility in the management of the health workforce. 

From the beginning of the pandemic until April 2021, the NHS had hired 83,180 new personnel: 25.7% were physicians and 38.5% were nurses [70]. However, the great majority of the staff had temporary contracts and there was therefore a need for qualitative changes in health workforce policy [65]. In March 2020, the government introduced the USCA (Unità Speciali di Continuità Assistenziale) with the statutory duty to manage COVID-19 patients treated at home. USCA are NHS units formed of physicians (usually with a specialization in general medicine), nurses, and other HCWs. The use of employment contracts for general practitioners (GPs) represents an important departure from previous staff policies in Italy, where GPs have always been independent workers.

At the organizational level, significant action was taken to re-organize emergency units and hospitals, not only to treat COVID-19 patients but also to prevent the spread of the infection within these services, as happened in the first months of the pandemic. During the same period, there was evidence of a shortage of PPE, lacking especially in nursing homes [71]. Elaborating on official data, some studies highlighted that, from 1st January to 31st May 2020 the probability to be infected was 15 to 20 times higher for HCWs compared to the rest of the population, with the highest levels reached by nurses [72]. The national government also introduced a “Corona bonus” for physicians, nurses, and other health professional staff.

Gender equality was largely ignored during the pandemic. One exception was the compensation introduced in March 2020 for parents, who needed to work at a time when childcare services and schools were closed; this was raised to EUR 2000, if parents were health or social care workers. No specific policy was adopted for migrant HCWs. In May 2020, the national government promoted regulation of undeclared or irregular migrant domestic carers, home care, and rural workers. A total of 207,542 applications were presented: 85% of the applications concerned domestic work and home care workers. However, the evaluation of the applications is proceeding very slowly [73].

### 3.3. A Cross-Country Comparative Overview of Major Results

Major results from our four explorative country case studies are summarized below using an illustrative table (Table 3). The comparative overview is guided by the conceptual framework applied to our case studies (Table 2) but also considers results from the quantitative investigation (Table 1). 

**Table 3:** A cross-country comparative overview of major results.

Categories	Comparative Results
Health policy responses to COVID-19	Denial of the pandemic in Brazil (under past President Bolsonaro) with action taken mainly at a local/community level; strong lockdown and social distancing policies in Italy, more moderate in Germany and Canada; access to vaccination (in Brazil only after pressure on Bolsonaro government) and some financial support to mitigate the effects in all countries, strongest in Germany.
Public health sector and hospital sector	Well-established and experienced public health sector in Brazil, similar in Canada and Italy, but weak in Germany; the hospital sector shows the opposite picture with a very strong position in Germany and weaker in Brazil, Canada, and Italy.
Health workforce and workplace conditions	High levels of death of HCWs, in particular nurses, poor PPE and training support in Brazil; lower risk of death and better PPE protection in Germany and Canada; more mixed in Italy with poor protection especially during the first wave. Staffing levels and HCWF composition vary strongly, with Brazil placed at a comparably lower and Germany at upper levels and Italy and Canada in-between. However, workplace stress worsened, shortages were an important problem in all countries, policy attention was overall poor, and effective solutions were lacking.
Individual actors, mental health conditions	Poor mental health of HCWs; lack of attention especially during the first waves of the pandemic; overall poor mental health support in all countries.
Gender equality, the situation of women HCWs	Lack of attention to gender equality and the needs of women HCWs; especially nurses, who were hit most severely; exacerbation of social inequalities.
The situation of migrant HCWs	Lack of attention to migrant HCWs and their needs; exacerbation of social inequalities.

Sources: authors’ own table.

## 4. Discussion

We argued that the health workforce is the “heart and soul” of every healthcare system [1] and that unpacking of health policy and population health must urgently include HCWF policy and how this affects the individual HCWs. Comparative research may contribute empirical evidence of existing governance gaps and systemic weaknesses, that threaten both the health and wellbeing of HCWs and the capacity of the HCWF to respond effectively to the crisis. We illustrated a descriptive comparison, drawing on basic categories of health systems related to our topic. So, what can be learned from the comparative exercise? The strength of this approach is its capacity to quickly provide an overview based on easily accessible and standardized items, for instance, provided by the OECD [46]. However, there are also important limitations and blind spots of descriptive comparison and quantifying indicators. 

A qualitative explorative approach may provide deeper insights [43]. Our country case studies reveal that different health systems often responded in largely similar ways to solve the health workforce challenges of the COVID-19 pandemic and that these policy responses show similar problems (Table 3). Major health policy weaknesses and failures include, among others, a lack of attention to gender equality policies and the situation of women HCWs during the pandemic, as well as to the needs of migrant HCWs. Effective stress prevention and management of an increased workload were also poorly developed and may have a negative impact on the mental health and wellbeing of the HCWs and, more generally, on health workforce recruitment and retention. The findings are supported by the literature [2,7,23,24]. They move beyond the health workforce and challenge comparative health policy. 

Rethinking approaches and categories of comparative health policy may open “windows of opportunity” for paying greater attention to health workforce issues. As an intersectoral policy arena and multidisciplinary research field, health workforce research lies across the classic comparative health policy categories of “governance”, “finance”, and “provision” and may thus contribute to innovative approaches and policy learning. We seek to illustrate this opportunity by highlighting some policy recommendations emerging from our explorative country cases.

As observed in other sectors, the pandemic worked as a focal glass. Existing problems of shortages of HCWs and poor work conditions became more visible [74]. The situation of the HCWs seemed to be worst in the LTC sector, e.g., HCWs in LTC were less well equipped with PPE compared to the hospital sector. Furthermore, health systems sought to increase staffing levels, but no country had plans in place for a sustainable and resilient health workforce, and no country responded adequately to the needs of the HCWs [7,9,16,75]. The findings call for greater attention to the actors and the conditions at the micro-level of the health workforce.

Across our country cases, there is strong evidence that the pandemic reinforced social inequalities. Gender and racial inequalities increased in the health workforce; migrant HCWs often faced higher risks of contracting COVID-19 than domestic HCWs. Similar problems are reported in the literature from a wide range of countries [7,9,16,50,76]. They cannot be solved at the organizational level, but need comprehensive action from the government and society.

In relation to governance, countries combined strategies at different levels. Even strongly decentralized and federalist healthcare systems mobilized national governance capacities [60]. At the same time, healthcare organizations, as well as professional associations and individual HCWs, played a major role in developing ad hoc solutions [2]. These new governance combinations are a “natural experiment” that should be used for policy learning. During the pandemic, major governance responsibilities were shifted to the organizational level. This has led to a situation, where workload and stress increased for lower-middle management and frontline HCWs. One important lesson is that health workforce governance needs a systematic and comprehensive approach, as problems cannot be solved as workplace or labor market issues at an operational level.

There are also some differences between countries. Most importantly, the Brazilian case (under the President Bolsonaro government) illustrates most clearly that politics matter. The empirical findings highlight that populist politics and an authoritarian President may outflank institutional conditions, creating high risks for HCWs and population health, and exacerbating social inequalities [10]. This supports Greer et al., arguing that “if some good comes out of the pandemic of 2020…perhaps it will be in showing not just that politics matter to health, but how, when and why they matter” (p. 4) [23].

Finally, the results suggest that comparative health workforce policy cannot be reduced to a health labor-market indicator, which is simply added to an existing framework for comparison; different health workforce staffing levels may create similar policy responses (and failures) and cause similar problems for the HCWs (Table 3). Health workforce policy must be analyzed in the context of institutions, governance, and politics and considered as an individual field of comparative health policy. Our multi-level and trans-sectoral governance approach may provide a springboard for further theoretical and empirical investigation, including greater attention to politics and actors.

## 5. Conclusions

This research set out to bring health workforce policy into comparative health policy research. We argued that healthcare workers are crucial for healthcare systems resilience and population health, and that health policy must be held accountable for their protection during the COVID-19 pandemic. We discussed two different comparative approaches, including a descriptive approach based primarily on health system categories and a qualitative explorative case study design, and illustrated their empirical capacity. Our results highlight research and policy recommendations.

- Health policy research must pay greater attention to health workforce policy and healthcare workers. Our comparative research investigation has illustrated the benefits of an integrated governance approach and a country case-study design based on various categories beyond health system typologies;
- Similar health workforce problems across countries highlight essential problems of pandemic policy and politics that may threaten HCWs and health system resilience. There is an urgent need for health policy to respond more effectively to the needs of individual HCWs and prevent gender, racial, and other inequalities during a major global health crisis.

## Figures and Tables

**Table 1:** A multi-level governance tool for comparing COVID-19 health workforce policy.

Substance of Governance	Assessment Categories
System level	- integration of education, healthcare, and labor market systems - financial compensation and bonuses - HCW vaccination program - surveillance and monitoring of COVID-19 incidence and deaths among HCW
Sector level	- integration of sectors, role of the hospital sector - public health roles, leadership, and adaption to new tasks
Organizational/ occupational level	- innovation in collaboration, skill-mix, and team approaches - coordination and leadership - training programs and public health competencies - personal protective equipment (PPE) and implementation of vaccination policy, surveillance programs - support for mental health during the pandemic, social support structures
Sociocultural dimension	- focus on HCW gender equality and HCW minority groups (or race) during COVID-19 (as selected illustrative examples of sociocultural issues)

Source: adapted from [7].

**Table 2:** Health system and health workforce indicators in four selected country cases: a comparative descriptive overview of basic indicators.

Categories	Brazil	Canada	Germany	Italy
**Health system** Governance	Unified health system (SUS) with free access to health services, federative coordination, and responsibilities devolved to government and municipalities.	Beveridge-type health system, decentralized federalism with most responsibilities devolved to provinces and territories.	Social Health Insurance (SHI) system, joint SHI self-administration, decentralized, federalist, responsibilities devolved to states, communities, and corporatist actors.	National Health Service (NHS) system, decentralized, responsibilities for management of healthcare services devolved to regions.
Finance	Funded by taxes, with mandatory contribution of 15–22% of municipal, state and federal budgets.	Funded mainly by income tax payments; 70% public, 30% private financing.	Funded mainly by employer and employee contributions with additional tax and private sources.	Funded mainly by taxes; about 75% public, 25% private financing.
Total health expenditure, all providers, %GDP *	9.6	11.78	12.8	9.5
Provision	Public and universal provision; additional voluntary private health insurance available.	Public provision of medical and hospital care; private mix for long term care, re-adaptation, and dental care.	Public provision with private mix (strong in long-term care), but joint SHI regulation	Public provision with significant private mix in hospital and outpatient care.
Hospital beds per 1000 population *	2.47	2.55	7.82	3.19
**Healthcare workforce** Total health and social employment density *	no data	55.48	74.0	33.63
Physician density *	2.15	2.77	4.53	4.13
Nurse density *	1.55 (7.43 ^#^)	10.06	12.06	6.26
Professional carers density *	no data	6.3	7.57	10.25
COVID-19 ** Infection rate, % of population	9.71	3.94 *	4.69	7.45
Death rate, % of cases	2.79	1.81	2.36	2.86
Vaccinated % of population, Fully vaccinated	70.63 42.46	76.81 70.83	67.49 63.98	74.89 67.90

Sources: authors’ own table. * OECD [46]; 2021 or nearest year; for workforce data: per 1000 population, head counts, practicing; accessed 7 February 2023. ^#^ Brazil, nurse density data provided by the Brazilian Government were significantly higher. ** Corona, Our World in Data [47]; cumulative confirmed cases per 47 million people, data as per 30 September 2021.

## Data Availability

All relevant information is cited and provided in the list of references.
